# Protective Effects of *Inula japonica* Leaf Extract Against PM_10_-Induced Oxidative Stress in Human Keratinocytes

**DOI:** 10.3390/cimb47080639

**Published:** 2025-08-09

**Authors:** Yea Jung Choi, So-Ri Son, Sullim Lee, Dae Sik Jang

**Affiliations:** 1 College of Korean Medicine, Gachon University, Seongnam 13120, Republic of Korea; domdada22@gachon.ac.kr; 2 College of Pharmacy, Kyung Hee University, 26 Kyungheedae-ro, Dongdaemun-gu, Seoul 02453, Republic of Korea; allosori@khu.ac.kr; 3 Department of Life Science, College of Bio-Nano Technology, Gachon University, Seongnam 13120, Republic of Korea

**Keywords:** human normal keratinocytes, particulate matter, *Inula japonica*, oxidative stress, skin disorder

## Abstract

This study aimed to evaluate the protective effects of *Inula japonica* leaf extract against PM_10_-induced oxidative stress in normal human keratinocytes. Keratinocytes were pretreated with various concentrations of *Inula japonica* leaf extract and subsequently exposed to PM10. Cell viability, ROS production, gene and protein expression (qRT-PCR and Western blot), and UHPLC-MS profiling were assessed. Network pharmacology analysis was conducted using database-predicted compounds of *Inulae Flos*. The extract significantly reduced PM_10_-induced ROS generation and restored the expression of epidermal barrier-related genes such as loricrin. It also inhibited phosphorylation of MAPKs (ERK, p38) and modulated apoptotic and inflammatory markers including Bax, p53, MMP-9, and COX-2. UHPLC-MS analysis identified eight compounds not previously reported in our earlier study, which may contribute to the extract’s protective effects. *Inula japonica* leaf extract exerts protective effects against PM_10_-induced skin damage by reducing oxidative stress and inflammation in keratinocytes. These findings support its potential as a therapeutic candidate for pollution-related skin disorders.

## 1. Introduction

Fine dust is categorized by particle size into particulate matter ≤ 0.1 μm, ≤ 2.5 μm, and ≤ 10 μm in diameter (PM_0.1_, PM_2.5_, and PM_10_), with PM_10_ encompassing all three fractions of particulate matter. These particles can accumulate on the skin surface, promoting oxidative stress through reactive oxygen species (ROS) generation and inducing the expression of pro-inflammatory mediators [1,2].

Keratinocytes are the predominant cell type in the epidermis and play critical roles in maintaining the skin barrier and regulating inflammatory responses [3,4]. When exposed to external stimuli, such as fine dust or ultraviolet radiation, keratinocytes may become damaged, leading to the release of inflammatory mediators and abnormal cellular responses, including apoptosis [5,6].

ROS generation induced by PM_10_ exposure activates key intracellular signaling pathways, such as mitogen-activated protein kinases (MAPKs) and nuclear factor kappa B (NF-κB), ultimately increasing the expression of inflammatory cytokines and pro-apoptotic factors, including tumor protein p53 (p53) and Bcl-2-associated X protein (Bax) [7,8]. The resulting increase in oxidative stress and inflammation contributes to skin barrier disruption, the formation of freckles and wrinkles, and may promote the development of various inflammatory skin conditions [9,10]. As oxidative stress and related signaling pathways are involved in PM_10_-induced keratinocyte damage, which has been linked to various skin conditions, this mechanism may contribute to disease progression [11,12].

Various natural compounds, including eckol, punicalagin, epigallocatechin gallate (EGCG), Korean propolis, and ginsenoside Rb1, have been reported to mitigate PM-induced oxidative stress in keratinocytes. However, these compounds are not yet approved for clinical use, and further studies are necessary to assess their therapeutic potential in preventing PM-induced oxidative skin damage [13,14,15,16].

Futhermore, PM exposure has been implicated not only in respiratory conditions such as asthma and chronic obstructive pulmonary disease but also in cardiovascular disease, systemic oxidative stress, and cutaneous inflammatory disorders, including acne [17,18,19].

*Inula japonica* Thunb., a perennial herb native to East Asia, has traditionally been used in herbal medicines. Previous studies have demonstrated that *Inula* flowers possess anti-inflammatory, anti-allergic, and anti-obesity properties [20,21,22,23]. Our recent study also identified caffeic acid derivatives from *Inula japonica* leaves that confer protective effects against tumor necrosis factor-α (TNF-α)-induced damage in human dermal fibroblasts [23]. Despite these findings, the pharmacological potential of *Inula japonica* leaf extract, particularly in the context of psoriasis, remains unexplored. Given the chemical heterogeneity among different plant parts, it is essential to evaluate the bioactivity of leaf-derived constituents independently of that of flower-derived components.

Accordingly, this study aimed to evaluate the therapeutic potential of Inula japonica leaf extract prepared using 30% ethanol in mitigating PM_10_-induced oxidative stress in normal human keratinocytes. Using a PM_10_-stimulated in vitro model, we assessed the protective effects of the extract and explored its mechanism of action through network pharmacology and experimental validation.

To the best of our knowledge, this is the first study to investigate the protective properties of *Inula japonica* leaves using a keratinocyte-based PM_10_ exposure model. These findings provide novel insights into the potential utility of this underexplored plant part for treating pollution-related skin inflammation and may guide future therapeutic development of natural products.

## 2. Materials and Methods

### 2.1. Materials

Dulbecco’s Modified Eagle Medium (DMEM; Corning Inc., Corning, NY, USA), EZ-Cytox Cell Viability Assay Kit (Daeil Lab Service, Seoul, Republic of Korea), 2′,7′-dichlorodihydrofluorescein diacetate (DCFDA; Sigma-Aldrich, St. Louis, MO, USA; Cat. No. D6883), Bicinchoninic Acid (BCA) Assay Kit (Thermo Fisher Scientific, Waltham, MA, USA; Cat. No. 23227), dimethyl sulfoxide (DMSO; Thermo Fisher Scientific, Waltham, MA, USA), QuantStudio 3 system (Applied Biosystems, Thermo Fisher Scientific, Waltham, MA, USA), and phosphate-buffered saline (PBS; Takara Bio Inc., Otsu, Japan) were used in this study.

### 2.2. Plant Material and UHPLC-Orbitrap-MS Analysis

The leaves of flowering *Inula japonica* growing along the Tancheon stream were collected in July 2023 at Gangnam-gu, Seoul, Korea (37°29′57″ N, 127°04′59″ E). The plant material was authenticated by Prof. Dae Sik Jang, and a voucher specimen (IN-JA4-2023) was deposited at the College of Pharmacy, Kyung Hee University, Seoul, Korea. After collection, the leaves were dried in a dryer at 50 °C. The dried *I*. *japonica* leaves (10.0 g) were extracted three times with 100 mL of 30% EtOH at room temperature for 24 h, followed by filtration and concentration (yield: 18.1%).

For further identification of the 30% EtOH extract of *I*. *japonica* leaves, a UHPLC-Orbitrap-MS analysis was performed using a previously described method [23]: The dried extract was dissolved in 50% MeOH (50 mg/mL), filtered, and subjected to UHPLC-Orbitrap-MS analysis using a HALO C18 column and a linear gradient elution system (from 3%B to 50%B) with 0.1% formic acid in water (A) and acetonitrile (B) as mobile phases. The MS parameters were optimized for high-resolution analysis, with the spray voltage set at −3000 V, capillary temperature at 320 °C, and probe heater temperature at 400 °C. Full MS spectra were acquired at a resolution of 70,000 (scan range: *m*/*z* 200–2000), and data-dependent MS/MS spectra were obtained at a resolution of 17,500 using stepped collision energies of 10, 30, and 40 eV.

Further identification of constituents from INJA was conducted through MS/MS analysis using in silico fragmentation analysis by MS-FINDER software (ver. 3.60). The precursor ions were processed with the formula finder to predict possible molecular formulas based on accurate *m*/*z* values and isotope pattern matching. Subsequently, the Structure Finder module was used to generate candidate structures by referencing chemical databases and biosynthetic rules.

For the network pharmacology analysis, the constituents of *Inulae Flos* were searched using the Traditional Chinese Medicine System Pharmacology Database and Analysis Platform (TCMSP; https://tcmsp-e.com/tcmsp.php, accessed on 25 October 2024). Among the active compounds, only those with an oral bioavailability of 30% or higher and a drug-likeness score of 0.18 or higher were selected.

### 2.3. Selection of Proteins Associated with Active Compounds

Target proteins associated with the selected active compounds were retrieved from the Search Tool for Interacting Chemicals (STITCH) database (http://stitch.embl.de/, accessed on 10 November 2024), restricted to *Homo sapiens*. Only protein–compound interactions with a confidence score ≥ 0.400 were included in the analysis.

### 2.4. Collection of Psoriasis-Related Genes

Genes associated with both psoriasis and *Inula japonica* were obtained from the GeneCards database (https://www.genecards.org/, accessed on 20 November 2024). Common overlapping genes were selected for further analyses.

### 2.5. Construction of Protein–Protein Interaction (PPI) Network

The protein–protein interaction network of the overlapping genes was constructed using the Search Tool for the Retrieval of Interacting Genes/Proteins (STRING) database (https://string-db.org/, accessed on 19 November 2024).

### 2.6. Gene Ontology (GO) and Kyoto Encyclopedia of Genes and Genomes (KEGG) Pathway Analysis

Gene Ontology (GO) and Kyoto Encyclopedia of Genes and Genomes (KEGG) pathway enrichment analyses were performed using Clustering with Gene Ontology (ClueGO) v2.5.10 in Cytoscape v3.10.2. These analyses were conducted to elucidate the potential mechanisms of *Inulae Flos* in psoriasis based on the shared target proteins between psoriasis and *Inulae Flos*.

### 2.7. Cell Culture and Sample Preparation

Normal human keratinocytes were purchased from PromoCell GmbH (Heidelberg, Germany; Cat. No. C-12006) and cultured in DMEM supplemented with 10% fetal bovine serum (FBS; Atlas Biologicals, Fort Collins, CO, USA) and 1% penicillin (Gibco, Thermo Fisher Scientific, Waltham, MA, USA). The cells were subcultured every three days. *Inula japonica* extract was dissolved in DMSO (final concentration of DMSO was 0.1%) to prepare a stock solution of 100 mg/mL. PM_10_ (ERM^®^, European Commission, Joint Research Centre, Geel, Belgium, Cat. No. ERM-CZ120) was dissolved in PBS to prepare a 100 mg/mL stock solution.

### 2.8. Cell Viability

Human keratinocytes were seeded into 96-well plates at a density of 1 × 10^4^ cells/well. After 24 h, the medium was replaced with serum-free DMEM to synchronize the cell cycle. Another 24 h later, cells were treated with various concentrations (1, 3, 10, 30, and 100 μg/mL) of *Inula japonica* leaf extract. After 24 h of treatment, EZ-Cytox reagent diluted 10% in DMEM was added, and the cells were incubated for 1 h. Absorbance was measured at 450 nm using a microplate reader (Agilent Technologies, Santa Clara, CA, USA). This assay is based on the conversion of a reagent by mitochondrial enzymes in viable cells, resulting in a color change proportional to cell viability.

### 2.9. ROS Inhibition Assay

Human keratinocytes were seeded into black 96-well plates at a density of 1 × 10^4^ cells/well. After 24 h, cells were synchronized by replacing the medium with serum-free DMEM. After pretreatment with various concentrations (10, 30, and 100 μg/mL) of *Inula japonica* leaf extract for 1 h, cells were incubated with 10 μM DCFDA in methanol for 30 min and subsequently exposed to 100 μg/mL PM_10_ for an additional 1 h. After incubation, the wells were washed twice with PBS, and the fluorescence intensity was measured at 485/535 nm (excitation/emission) using a microplate reader (Agilent Technologies). This assay detects intracellular reactive oxygen species by measuring the fluorescence of DCFDA, which is oxidized to a fluorescent compound in the presence of ROS.

### 2.10. Western Blot

Human keratinocytes were seeded in 6-well plates at a density of 3 × 10^5^ cells/well. After 24 h, the medium was replaced with serum-free Dulbecco’s Modified Eagle Medium (DMEM) for cell cycle synchronization. The cells were then treated with varying concentrations (10, 30, and 100 μg/mL) of *Inula japonica* leaf extract. One hour later, PM_10_ (particulate matter with a diameter ≤ 10 μm, 100 μg/mL) was added to the cells. Cells were harvested using Radioimmunoprecipitation Assay (RIPA) buffer containing a protease inhibitor cocktail (Sigma-Aldrich, St. Louis, MO, USA) and a phosphatase inhibitor cocktail (Roche, Basel, Switzerland) either 30 min later for MAPKs phosphorylation analysis (extracellular signal-regulated kinase; ERK, phosphorylated extracellular signal-regulated kinase; p-ERK, c-Jun *N*-terminal kinase; JNK, phosphorylated c-Jun *N*-terminal kinase.; p-JNK, p38 mitogen-activated protein kinase; p38, and phosphorylated p38 mitogen-activated protein kinasep-p38) or 24 h later to assess the expression of Bax, p53, matrix metalloproteinase-9 (MMP-9), cyclooxygenase-2 (COX-2), heme oxygenase-1 (HO-1), phosphorylated Protein Kinase B (p-Akt), and glyceraldehyde-3-phosphate dehydrogenase (GAPDH). Primary antibodies (Cell Signaling Technology, Danvers, MA, USA) were diluted 1:1000 in Tris-buffered saline with Tween 20 (TBST) for all Western blot experiments. Western blotting allows for the detection and quantification of specific proteins by separating them based on size, transferring them to membranes, and using antibodies to visualize target proteins.

Protein concentration was determined using a Bicinchoninic Acid (BCA) assay. Equal amounts of protein were separated by sodium dodecyl sulfate-polyacrylamide gel electrophoresis (SDS-PAGE) and transferred to Polyvinylidene Difluoride (PVDF) membranes. Membranes were blocked with 5% skim milk in PBS, incubated with primary antibodies overnight at 4 °C, followed by secondary antibody incubation for 1 h at room temperature. Protein bands were visualized using Enhanced Chemiluminescence (ECL) and detected using a ChemiDoc imaging system (Bio-Rad, Hercules, CA, USA). Band intensities were quantified using the ImageJ software (Version 1.51J; NIH, Bethesda, MD, USA).

### 2.11. qRT-PCR Assay

Human keratinocytes were seeded in 6-well plates at a density of 3 × 10^5^ cells/well. After 24 h, the medium was replaced with serum-free DMEM, and *Inula japonica* extract was administered at various concentrations (10, 30, and 100 μg/mL). One hour later, PM_10_ (100 µg/mL) was added, and the cells were incubated for 24 h. Cells were harvested using the RLT buffer.

Total RNA was extracted using the Qiagen RNA Mini Kit (Qiagen, Hilden, Germany), and the RNA concentration was measured using a NanoDrop spectrophotometer (ACTGene, Piscataway, NJ, USA). Complementary DNA (cDNA) was synthesized using a cDNA synthesis kit. Quantitative Real-Time PCR (qRT-PCR) was performed using SYBR Green reagent (Applied Biosystems, Thermo Fisher Scientific, Waltham, MA, USA) on a QuantStudio 3 system. Primer sequences are listed in Table 1, and β-actin was used as the internal control. Relative gene expression was calculated using the comparative ΔΔCt method following the manufacturer’s instructions. qRT-PCR quantifies gene expression by amplifying specific cDNA targets and measuring the fluorescence intensity proportional to the amount of the amplified product. *Cytosolic phospholipase A2* (*cPLA2*), *keratin 16* (*KRT16*), *filament aggregating protein* (*Filaggrin*), *loricirin*, and *involucrin* were measured.

### 2.12. Statistical Analysis

Statistical analyses were performed using GraphPad Prism 8 (GraphPad Software, San Diego, CA, USA). Data are presented as mean ± standard error of the mean (SEM) based on two or three independent experiments. Statistical significance was determined using one-way analysis of variance (ANOVA) at a significance level of *p* < 0.05. Tukey’s post hoc test was used for multiple group comparisons.

## 3. Results

### 3.1. LC-MS-Based Identification of Flavonoids in Inula japonica Leaves

**Figure 1:**
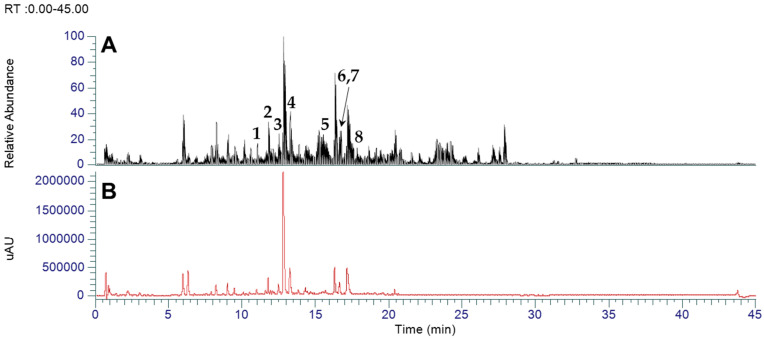
UHPLC-MS chromatograms of INJA: (**A**) total ion chromatogram (TIC; negative) of INJA and (**B**) diode array detector (DAD) chromatogram of INJA. Peak numbers displayed in the TIC correspond to the profiling results in Table 2.

To accurately identify the principal constituents from the 30% EtOH extract of *I*. *japonica* leaves (INJA), UHPLC-Orbitrap-MS analysis was conducted using a previously described method and reference standards (Figure 1) [23]. In our previous study, we identified caffeoylglucaric acids and caffeoylquinic acids as major components of INJA. However, as flavonoids and sesquiterpenes have also been frequently reported as key constituents of *I. japonica* in TCMSP, we determined whether these compound classes were also present in our extract. Eight additional flavonoids were identified in the *I*. *japonica* leaf extract that were not reported in previous studies (Table 2).

### 3.2. Effects of Inula japonica Leaf Extract on Cell Viability and Intracellular ROS Generation in PM_10_-Induced Human Keratinocytes

**Figure 2:**
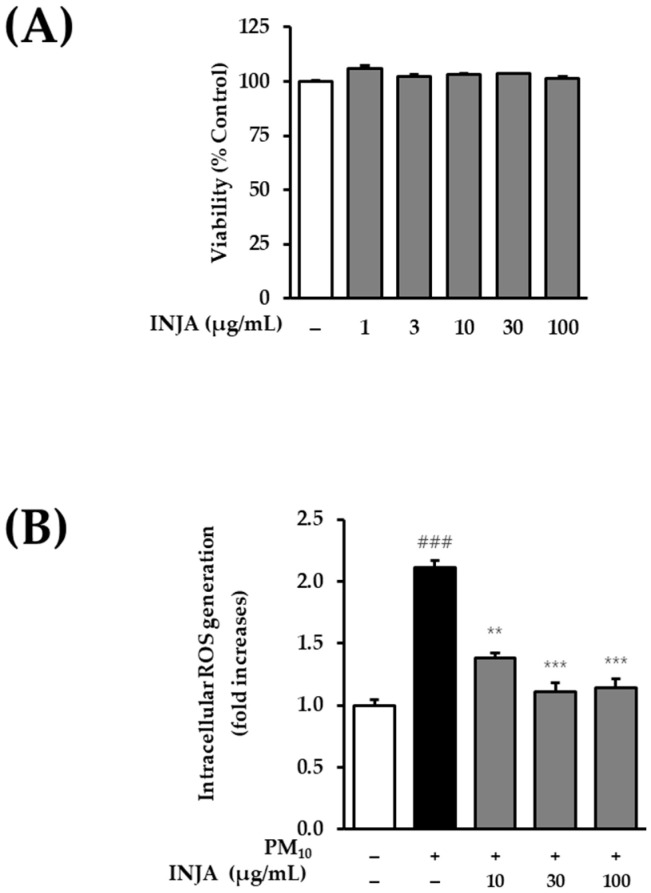
Effects of *Inula japonica* leaf extract on cell viability and PM_10_-induced ROS generation in normal human keratinocytes (NHKs). (**A**) Cell viability following treatment with various concentrations of *Inula japonica* leaf extract. (**B**) Intracellular ROS levels in PM_10_-treated keratinocytes with or without *Inula japonica* extract. Data are expressed as mean ± SEM from three independent experiments. ^###^ *p* < 0.001 vs. untreated group; ** *p* < 0.01, *** *p* < 0.001 vs. PM_10_-treated group.

As shown in Figure 2A, a cytotoxicity assay was performed to determine the non-toxic concentration range of *Inula japonica* leaf extract for subsequent experiments. The results demonstrated that the extract did not exhibit cytotoxicity at any of the tested concentrations in normal human keratinocytes.

In Figure 2B, PM_10_ exposure significantly increased intracellular ROS levels to 2.14 ± 0.05 (^###^ *p* < 0.001) compared to the untreated group. However, treatment with *Inula japonica* leaf extract significantly reduced ROS production in a concentration-dependent manner, with ROS levels measured at 1.38 ± 0.04 (** *p* < 0.01), 1.11 ± 0.07 (*** *p* < 0.001), and 1.14 ± 0.08 (*** *p* < 0.001) at concentrations of 10, 30, and 100 µg/mL, respectively.

### 3.3. Effects of Inula japonica Leaf Extract on Bax and p53 Protein Expression in PM_10_-Induced Human Keratinocytes

**Figure 3:**
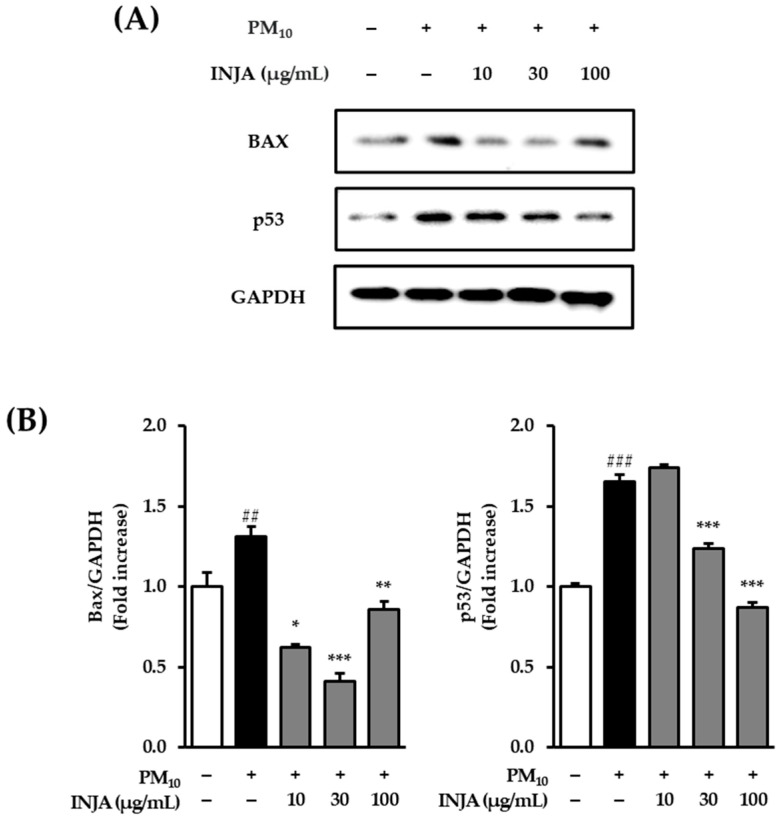
Effects of *Inula japonica* leaf extract on PM_10_-induced Bax and p53 protein expression in normal human keratinocytes (NHKs). (**A**) NHKs were treated with 10, 30, and 100 μg/mL *Inula japonica* leaf extract for 1 h and then with 100 μg/mL PM_10_ for 24 h. Immunoreactive bands were analyzed by immunoblotting for BAX, p53, and GAPDH. (**B**) The expression levels of BAX and p53 were normalized to GAPDH and are presented as relative protein levels. The measuring results are represented as mean ± SEM of duplicated experiments (*n* = 3). ^##^ *p* < 0.01, and ^###^ *p* < 0.001 compared to the non-treated group; * *p* < 0.05, ** *p* < 0.01, and *** *p* < 0.001 compared to the PM_10_-induced group.

As shown in Figure 3, exposure to PM_10_ significantly increased the expression of the pro-apoptotic protein Bax to 1.31 ± 0.06 (*p* < 0.01) compared to the untreated control group. However, treatment with *Inula japonica* leaf extract attenuated Bax expression, reducing it to 0.62 ± 0.02 (*p* < 0.05) at 10 µg/mL, 0.41 ± 0.05 (*p* < 0.001) at 30 µg/mL, and 0.86 ± 0.05 (*p* < 0.01) at 100 µg/mL.

Similarly, PM_10_ exposure increased p53 protein expression to 1.65 ± 0.04 (*p* < 0.001). Treatment with *Inula japonica* extract significantly decreased p53 levels to 1.24 ± 0.03 (*p* < 0.001) at 30 µg/mL and 0.87 ± 0.03 (*p* < 0.001) at 100 µg/mL.

### 3.4. Effects of Inula japonica Leaf Extract on Phosphorylation of MAPKs Proteins in PM_10_-Induced Human Keratinocytes

**Figure 4:**
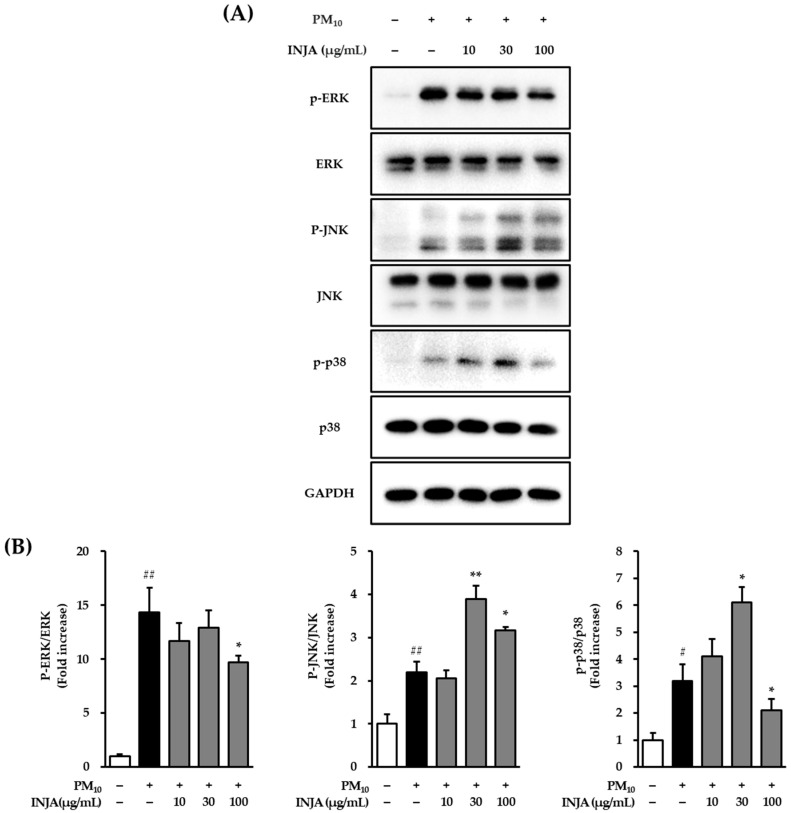
Effects of *Inula japonica* leaf extract on PM_10_-induced phosphorylation of MAPK proteins in human normal keratinocytes (NHKs). (**A**) NHKs were pretreated with 10, 30, or 100 μg/mL *Inula japonica* extract for 1 h, followed by stimulation with 100 μg/mL PM_10_ for 30 min. Protein expression was analyzed by Western blotting for total and phosphorylated forms of ERK, JNK, and p38, with GAPDH as a loading control. (**B**) Densitometric analysis of phosphorylated proteins (p-ERK, p-JNK, and p-p38) normalized to their respective total protein levels. Data are presented as mean ± SEM from three independent experiments (*n* = 3). ^#^ *p* < 0.05, ^##^ *p* < 0.001 vs. untreated group; * *p* < 0.05, ** *p* < 0.01 vs. PM_10_-treated group.

As shown in Figure 4, exposure to PM_10_ significantly increased phosphorylation of ERK by 14.37 ± 2.24-fold (*p* < 0.001) compared to the untreated control. Treatment with *Inula japonica* leaf extract significantly reduced ERK phosphorylation to 11.67 ± 1.71-fold (not significant) at 10 µg/mL, 12.89 ± 1.63-fold (not significant) at 30 µg/mL and 9.68 ± 0.67-fold (*p* < 0.05) at 100 µg/mL.

PM_10_ exposure also elevated JNK phosphorylation to 3.19 ± 0.62-fold (*p* < 0.001). Interestingly, treatment with *Inula japonica* leaf extract further increased JNK phosphorylation as follows: 3.89 ± 0.32-fold at 30 µg/mL (*p* < 0.01), and 3.17 ± 0.08-fold at 100 µg/mL (*p* < 0.05).

In contrast, PM_10_-induced phosphorylation of p38 increased to 1.58 ± 0.07-fold (*p* < 0.001). Treatment with *Inula japonica* leaf extract further increased p38 phosphorylation to 6.11 ± 0.58-fold at 30 µg/mL (*p* < 0.05), whereas treatment with 100 µg/mL *Inula japonica* leaf extract significantly reduced p38 phosphorylation to 2.10 ± 0.43-fold (*p* < 0.05).

### 3.5. Effects of Inula japonica Leaf Extract on MMP-9, HO-1, COX-2, and Phosphorylated AKT Expression in PM_10_-Induced Human Keratinocytes

**Figure 5:**
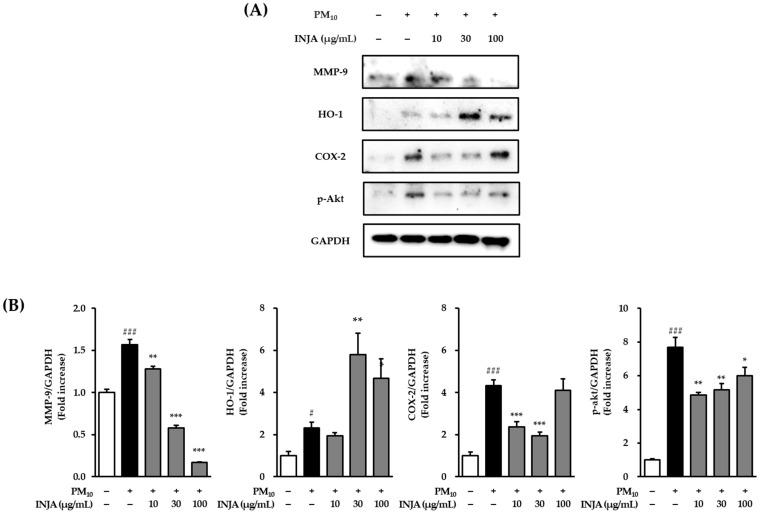
Effects of *Inula japonica* leaf extract on PM_10_-induced expression of MMP-9, HO-1, COX-2, and phosphorylated AKT (p-AKT) in normal human keratinocytes (NHKs). (**A**) NHKs were pretreated with 10, 30, or 100 μg/mL *Inula japonica* leaf extract for 1 h, followed by exposure to 100 μg/mL PM_10_ for 24 h. Immunoblotting was performed to detect MMP-9, HO-1, COX-2, p-AKT, and GAPDH. (**B**) Quantitative analysis of MMP-9, HO-1, COX-2, and p-AKT expression, normalized to GAPDH. Data are presented as mean ± SEM from three independent experiments (*n* = 3). ^#^ *p* < 0.05, ^###^ *p* < 0.001 vs. untreated group; * *p* < 0.05, ** *p* < 0.01, *** *p* < 0.001 vs. PM_10_-treated group.

As shown in Figure 5, exposure to PM_10_ significantly increased the expression of MMP-9 to 1.64 ± 0.07-fold (*p* < 0.001) compared to the untreated control group. Treatment with *Inula japonica* leaf extract suppressed MMP-9 expression in a dose-dependent manner, reducing it to 1.28 ± 0.09-fold (*p* < 0.001) at 10 µg/mL, 0.64 ± 0.07-fold (*p* < 0.001) at 30 µg/mL, and 0.21 ± 0.03-fold (*p* < 0.001) at 100 µg/mL. PM_10_ exposure also elevated HO-1 expression to 2.31± 0.29-fold (*p* < 0.05). Interestingly, treatment with 30 and 100 µg/mL of *Inula japonica* extract further increased HO-1 expression to 5.78 ± 1.03-fold (*p* < 0.01) and 4.68 ± 0.92-fold (*p* < 0.05), suggesting a potential cytoprotective effect.

In addition, COX-2 phosphorylation was significantly increased by PM_10_ to 4.34 ± 0.28-fold (*p* < 0.001). Treatment with *Inula japonica* extract reduced COX-2 phosphorylation to 2.36 ± 0.25-fold (*p* < 0.001) at 10 µg/mL and 1.95 ± 0.16-fold (*p* < 0.001) at 30 µg/mL. Furthermore, PM_10_ stimulation increased Akt phosphorylation to 7.67 ± 0.62-fold (*p* < 0.001), whereas treatment with *Inula japonica* extract significantly decreased p-AKT levels to 4.86 ± 0.14-fold (*p* < 0.01) at 10 µg/mL, 5.17 ± 0.38-fold (*p* < 0.01) at 30 µg/mL, and 6.00 ± 0.51-fold (*p* < 0.05) at 100 µg/mL.

### 3.6. Effects of Inula japonica Leaf Extract on cPLA2, KRT16, Filaggrin, Loricrin, and Involucrin mRNA Expression in PM_10_-Induced Human Keratinocytes

**Figure 6:**
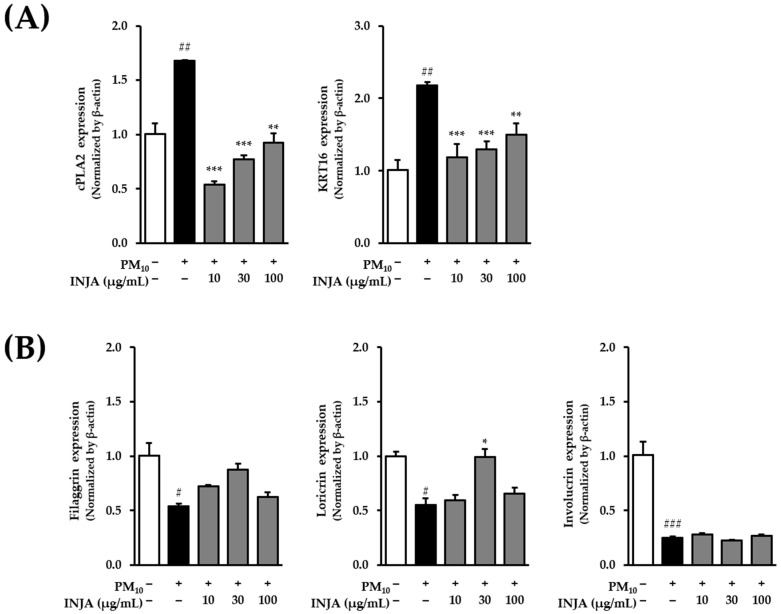
Effects of *Inula japonica* leaf extract on PM_10_-induced mRNA expression of *cPLA2*, *KRT16*, *Filaggrin*, *Loricrin*, and *Involucrin* in normal human keratinocytes (NHKs). (**A**,**B**) NHKs were pretreated with 10, 30, or 100 μg/mL *I. japonica* leaf extract for 1 h, followed by exposure to 100 μg/mL PM_10_ for 24 h. β-actin was used as a housekeeping gene. Gene expression levels in the PM_10_-treated groups were normalized to those in the untreated controls. Data are presented as mean ± SEM from two independent experiments (*n* = 2). ^#^ *p* < 0.05, ^##^ *p* < 0.01, ^###^ *p* < 0.001 vs. untreated group; * *p* < 0.05, ** *p* < 0.01, *** *p* < 0.001 vs. PM_10_-treated group.

PM_10_ (Figure 6) exposure significantly increased the mRNA expression of *cPLA2* to 1.68 ± 0.01-fold compared to that in the untreated control group (*p* < 0.01). Treatment with *Inula japonica* leaf extract reduced *cPLA2* expression in a dose-dependent manner to 0.54 ± 0.03-fold (*p* < 0.001) at 10 µg/mL, 0.80 ± 0.04-fold (*p* < 0.001) at 30 µg/mL, and 0.90 ± 0.10-fold (*p* < 0.01) at 100 µg/mL. Similarly, *KRT16* expression significantly upregulated PM_10_ treatment to 2.18 ± 0.06-fold (*p* < 0.01), whereas *Inula japonica* extract attenuated its expression to 1.18 ± 0.19-fold (*p* < 0.001) at 10 µg/mL, 1.30 ± 0.11-fold (*p* < 0.001) at 30 µg/mL, and 1.50 ± 0.20-fold (*p* < 0.01) at 100 µg/mL.

Furthermore, PM_10_ significantly downregulated *Loricrin* mRNA expression to 0.55 ± 0.06-fold (*p* < 0.01). However, treatment with 30 µg/mL of *Inula japonica* restored Loricrin levels to 1.00 ± 0.07-fold (*p* < 0.01), suggesting a recovery of epidermal barrier function. In contrast, *Inula japonica* extract had no significant effect on the mRNA expression of *Filaggrin* and *Involucrin*.

### 3.7. Database-Driven Screening of Inulae Flos

A total of 53 constituents of *Inulae Flos* were identified using the TCMSP database. Among them, 19 active compounds were selected based on oral bioavailability (OB) ≥ 30% and drug-likeness (DL) ≥ 0.18, as shown in Table 3.

### 3.8. Target Protein Prediction for Active Compounds of Inulae Flos Using the STITCH Database

**Figure 7:**
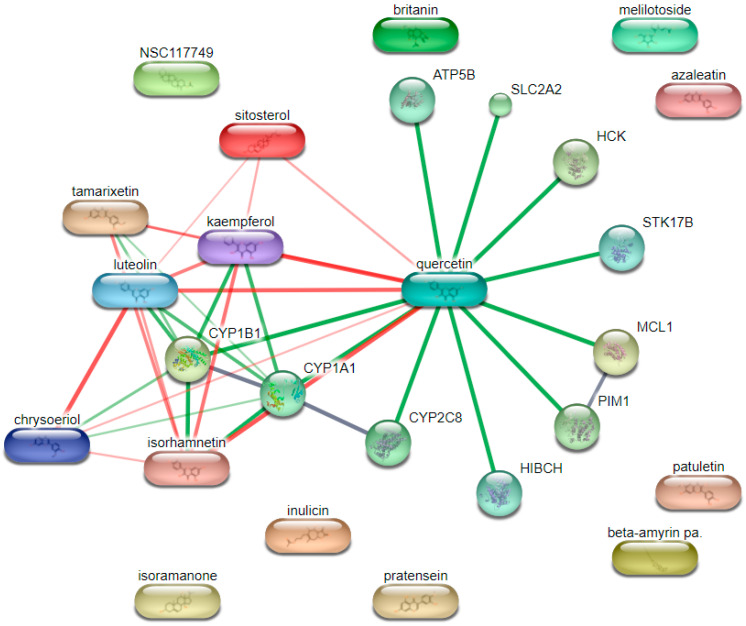
Predicted target proteins of *Inulae Flos* active compounds. Edge colors indicate the type of evidence supporting the interaction: green for curated database, red for text mining, and gray for experimental evidence.

Target proteins associated with the active compounds of *Inula japonica* were identified using the STITCH database, restricted to *Homo sapiens*. Compounds that were not present in the database were excluded from the analysis. A confidence score threshold of ≥0.4 was applied, which represents a moderate level of interaction reliability; a score closer to 1.0 indicates a stronger predicted association between a compound and its target protein. Based on this criterion, a total of 10 target proteins were identified (Figure 7) as follows: ATP synthase subunit beta (ATP5B), solute carrier family 2 member 2 (SLC2A2), hemopoietic cell kinase (HCK), serine/threonine kinase 17b (STK17B), myeloid cell leukemia 1 (MCL1), Pim-1 proto-oncogene (PIM1), 3-hydroxyisobutyryl-CoA hydrolase (HIBCH), Cytochrome P450 family 2 subfamily C member 8 (CYP2C8), Cytochrome P450 family 1 subfamily A member 1 (CYP1A1), and Cytochrome P450 family 1 subfamily B member 1 (CYP1B1). These proteins were selected for further analyses.

### 3.9. Identification of Common Targets and Network Analysis Between Inulae Flos Compounds and Psoriasis-Associated Genes

**Figure 8:**
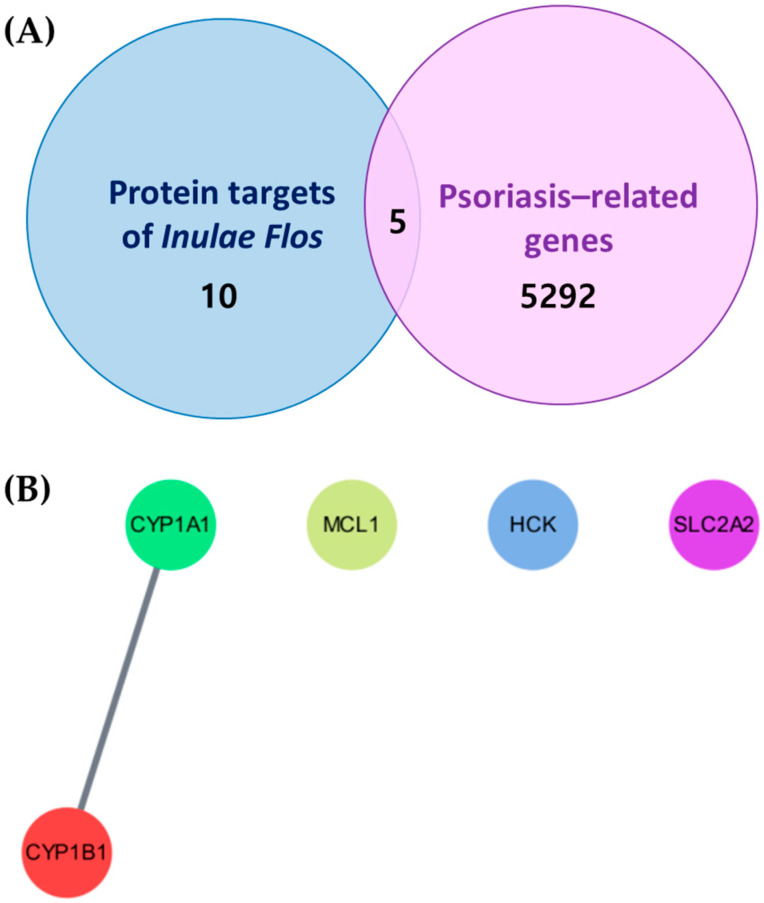
Common targets between *Inulae Flos* compounds and psoriasis-related genes: (**A**) Venn diagram illustrating overlapping target genes between *Inulae Flos* compound targets and psoriasis-associated genes, and (**B**) protein–protein interaction network of the five overlapping targets, constructed using STRING in Cytoscape.

As shown in Figure 8A, a total of 5292 psoriasis-related genes were retrieved from the GeneCards database. Comparative analysis between these genes and the 10 target proteins of *Inulae Flos* compounds revealed five overlapping genes shared by both datasets.

Subsequently, PPI network analysis of the five shared proteins was conducted using the STRING plugin in Cytoscape. As illustrated in Figure 8B, this analysis revealed a significant interaction between CYP1B1 and CYP1A1, suggesting that these proteins may represent key molecular targets through which *Inulae Flos* exerts its potential therapeutic effects in psoriasis.

### 3.10. Prediction of the Mechanism of Action of Active Compounds from Inulae Flos Using ClueGO

Pathway and functional enrichment analyses were performed using the ClueGO plugin in Cytoscape, based on Kyoto Encyclopedia of Genes and Genomes (KEGG) pathways and Gene Ontology (GO) terms. These analyses focused on the five shared target proteins identified in the previous step.

**Figure 9:**
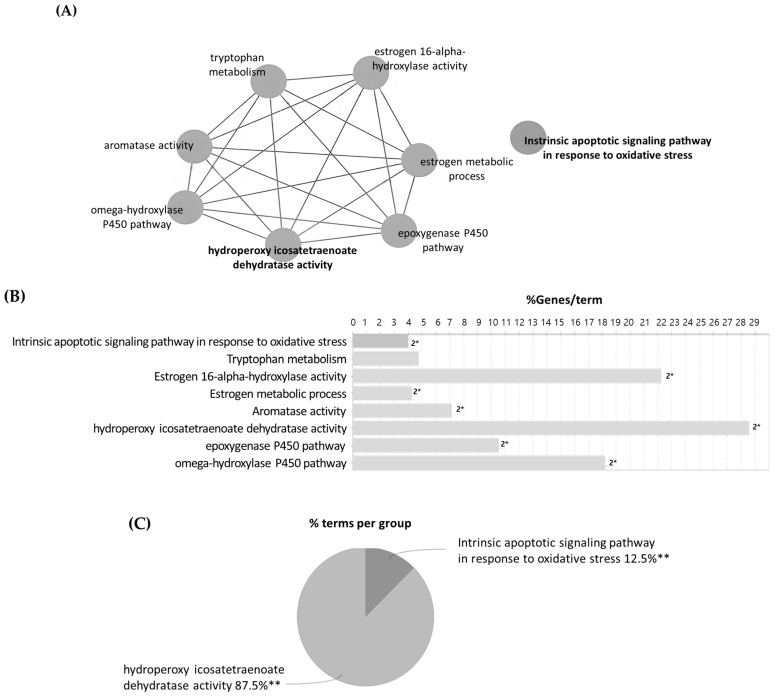
Functional enrichment analysis of overlapping targets using ClueGO: (**A**) functional network of five overlapping proteins between *Inulae Flos* compound targets and psoriasis-related genes; the number followed by * indicates the count of significant genes (* *p* < 0.05 compared to overall analysis). (**B**) percentage of genes associated with each enriched term; and (**C**) distribution of enriched terms by functional group. ** *p* < 0.01 vs. overall analysis group.

As shown in Figure 9A, the most significantly enriched terms were hydroperoxyl icosatetraenoate dehydratase activity and intrinsic apoptotic signaling pathway in response to oxidative stress. Figure 9B shows that hydroperoxyl icosatetraenoate dehydratase activity is associated with approximately 28–30 genes, indicating its broad involvement in various biological functions. In contrast, the intrinsic apoptotic signaling pathway in response to oxidative stress was linked to a smaller number of genes but displayed higher statistical significance based on the manually set threshold (*p* ≤ 0.05), suggesting a more specific and potentially critical role in the biological response. 

As illustrated in Figure 9C, among the major enriched terms, hydroperoxyl icosatetraenoate dehydratase activity accounted for 87.5%, while the intrinsic apoptotic signaling pathway in response to oxidative stress contributed 12.5%. This indicates that the former plays a predominant role in the functional classification of these intersection targets.

## 4. Discussion

PM_10_ is known to increase intracellular ROS levels in human keratinocytes, thereby activating MAPK signaling pathways and upregulating pro-inflammatory cytokines and matrix-degrading enzymes, such as Matrix Metalloproteinases (MMPs) [24,25]. All cell-based experiments were conducted under serum starvation conditions. Although serum starvation does not achieve molecular-level synchronization, it is a widely accepted method for arresting HaCaT keratinocytes at the G_0_/G_1_ phase in skin-related studies [26].

As shown in Figure 2, treatment with *Inula japonica* (INJA) extract significantly suppressed PM_10_-induced ROS production at all tested concentrations.

Furthermore, the data presented in Figure 3 indicate that INJA extract significantly reduced Bax protein levels at 10, 30, and 100 µg/mL and also decreased p53 expression at 30 and 100 µg/mL. Consistently, Figure 4 shows that INJA extract inhibited PM_10_-induced ERK phosphorylation at 10 and 100 µg/mL and p38 phosphorylation at 100 µg/mL. Interestingly, JNK phosphorylation was also increased.

Although JNK activation is commonly associated with pro-apoptotic and pro-inflammatory responses [27,28,29], the observed increase may represent a transient compensatory response to stress. However, this activation may not necessarily lead to enhanced cell death or inflammation. Further investigations of downstream targets, such as c-JUN and NF-κB, are required to clarify the biological implications of JNK activation [30].

As shown in Figure 5, INJA extract significantly reduced MMP-9 expression at all concentrations, suggesting a protective role through the suppression of ERK and p38 phosphorylation, ultimately leading to the downregulation of apoptotic markers (Bax and p53) and matrix-degrading enzymes.

Previous studies have supported these findings. Tart cherry extract inhibited ROS production and reduced ERK and p38 phosphorylation in PM_10_-exposed HaCaT cells, as well as suppressed p53, Bax, and cleaved caspase-3 expression [8]. Similarly, *Momordica cochinchinensis* extract suppressed ROS and MAPK activation in PM_10_-stimulated keratinocytes [31]. Other studies have demonstrated that in polycyclic aromatic hydrocarbon (PAH)-induced HaCaT cells, CYP1A1 inhibition reduces ROS levels and Bax expression, thereby attenuating apoptosis [32]. Similarly, CYP1B1 inhibition in benzo[a]pyrene-treated HaCaT cells reduced ROS and mitophagy, ultimately decreasing apoptotic cell death [33]. Furthermore, previous studies have demonstrated that Porphyra 334, isolated from *Porphyra yezoensis* water extract, effectively inhibits CYP1A1 expression and reactive oxygen species (ROS) generation in keratinocytes exposed to urban particulate matter [34].

These findings indicate that CYP1A1 and CYP1B1 play crucial roles in the regulation of ROS-mediated apoptosis. Although direct measurements of CYP1A1 and CYP1B1 were not conducted in this study, subsequent experiments examined ROS production and intrinsic apoptosis markers (Bcl-2 and Bax), which are closely related to CYP1A1/B1-regulated pathways.

When comparing the results of network pharmacology and in vitro experiments, INJA extract inhibited ERK and p38 phosphorylation, subsequently suppressing the intrinsic apoptosis pathway markers p53 and Bax. These effects are consistent with the pathways associated with CYP1A1 and CYP1B1, thereby validating our computational predictions.

Particulate matter 10 exposure also induces phosphorylation of AKT and upregulation of inflammatory mediators such as COX-2 and cPLA2 [35,36]. In Figure 5, INJA extract inhibited AKT phosphorylation at all tested doses and significantly suppressed COX-2 expression at 10 and 30 µg/mL. Moreover, *cPLA2* mRNA expression was significantly reduced (Figure 6), further supporting the anti-inflammatory effects of the treatment.

Interestingly, INJA extract upregulated HO-1 protein expression at 30 µg/mL concentration (Figure 5). While few studies have assessed HO-1 expression in PM_10_-treated keratinocytes, existing evidence links increased HO-1 expression with antioxidant protection in skin cells [37,38]. This finding supports the antioxidant effects observed in our study.

Excessive ROS disrupts keratinocyte hydration and structural integrity, leading to impaired skin barrier function and abnormal keratinocyte proliferation, which are key features of psoriasis. PM_10_ exposure has been shown to suppress skin barrier proteins, such as filaggrin, loricrin, and involucrin, while increasing KRT16 and Keratin 17(KRT17) expression, which are markers of hyperproliferative states [39,40]. In Figure 6, INJA extract significantly inhibited PM_10_-induced *KRT16* mRNA expression and restored loricrin expression at 30 µg/mL, indicating potential barrier-protective effects.

In summary, *Inula japonica* leaf extract effectively attenuated PM_10_-induced oxidative stress and inflammation in human keratinocytes. It inhibited the phosphorylation of ERK and p38, reduced the levels of apoptosis-related markers (p53 and Bax), suppressed the expression of MMP-9 and COX-2, and modulated AKT phosphorylation. The extract also enhanced antioxidant defenses via HO-1 and restored skin barrier markers, such as *loricrin*, while suppressing hyperproliferation-related *KRT16*. These in vitro results align well with network pharmacology predictions, supporting a multi-target mechanism of skin protection.

Natural products, including *Inula japonica*, contain a wide range of bioactive compounds that are expected to act on multiple molecular targets and signaling pathways rather than on a single target [41]. To better understand the multi-component, multi-target, and multi-pathway interactions, network pharmacology analysis was conducted. As summarized in Table 3, a total of 19 major compounds were identified, and 10 key target proteins were selected through compound–protein interaction network analysis (Figure 7). It should be noted that the analysis was based only on compounds listed in public databases; thus, components of *Inulae Flos* not included in these databases may have been omitted, representing a limitation of the study. Future research should utilize more comprehensive and updated databases to improve compound coverage and prediction accuracy. Furthermore, integration with psoriasis-related gene data revealed five overlapping genes, with CYP1A1 and CYP1B1 emerging as core targets (Figure 8). A previous study investigating the AhR-CYP signaling pathway in patients with exacerbated psoriasis reported a significant increase in serum CYP1A1 expression. CYP1B1 was also analyzed because of its involvement in this pathway [42]. Furthermore, CYP1A1 activity is significantly elevated in the blood cells of patients with psoriasis compared to healthy controls, suggesting its role in modulating psoriasis-related inflammation [43].

Gene Ontology (GO) and KEGG pathway enrichment analysis (Figure 9) revealed that these target genes were significantly associated with hydroperoxyl icosatetraenoate dehydratase activity and the intrinsic apoptotic signaling pathway. These findings suggest that *Inulae Flos* extract may modulate oxidative stress via enzymatic mechanisms involving hydroperoxyl icosatetraenoate dehydratase activity, while the intrinsic apoptotic signaling pathway may provide a targeted cellular defense. In a previous study, flavonoids such as patuletin, nepetin, and axillarin, isolated from *Inula britannica*, preserved antioxidant enzyme activities, including catalase, glutathione peroxidase (GSH-Px), and glutathione reductase (GSSG-R), in cortical neurons exposed to glutamate-induced oxida *Inulae Flos* tive stress, thereby suppressing lipid peroxidation [44]. Similarly, total flavonoids from *Inula japonica* (TFIJ) significantly inhibited lipid peroxidation markers, such as malondialdehyde (MDA) and myeloperoxidase (MPO), in an LPS-induced acute lung injury model [45]. In addition, sesquiterpene lactones, such as alantolactone and isoalantolactone, from *Inula helenium* have been reported to induce intrinsic apoptosis in thyroid cancer and oral squamous carcinoma cells through ROS generation, BAX/BCL-2 ratio modulation, and caspase-3 activation [46,47].

Although the network pharmacology analysis was based on compounds derived from *Inulae Flos* (the flowers of *Inula japonica*) as cataloged in the TCMSP database, there are currently no compound data available for the leaves of *Inula japonica* in this resource. Accordingly, the chemical profile of the leaf extract used in this study may differ from that of the flower-derived compounds listed in the database. Indeed, UHPLC-MS analysis revealed that many of the database-listed compounds were not detected in the actual leaf extract. This highlights a critical limitation of current phytochemical databases, which often lack specificity for different plant parts and their constituents. To improve the accuracy of target prediction and provide mechanistic insights, it is essential to develop a part-specific database focused on *Inula japonica* leaves.

As shown in Table 2, several major caffeoylquinic and caffeoylglucaric acids previously reported in *Inula japonica* were detected in the leaf extract, along with flavonoids. However, many sesquiterpenes predicted by the database were not observed in the LC-MS analysis, possibly due to limited extraction efficiency or environmental factors affecting metabolite production during plant growth.

Despite these differences, the leaf extract demonstrated activity consistent with the apoptosis pathway predicted by network pharmacology in a PM_10_-induced psoriasis-like environment. This suggests that the shared or structurally similar compounds between leaves and flowers may exert overlapping biological effects. Therefore, the current study provides a foundation for the future development of leaf-specific compound databases for *Inula japonica*.

In the present study, Inula japonica leaf extract was prepared using 30% ethanol, a solvent widely employed for extracting a broad range of phytochemicals due to its intermediate polarity. While extraction conditions were not the primary focus, the choice of solvent likely influenced the extract’s chemical composition and bioactivity. Optimization of extraction methods may therefore warrant further investigation in future studies.

A key strength of this study lies in its integrated approach, combining phytochemical profiling, in vitro validation using normal human keratinocytes, and network pharmacology analysis. This multi-dimensional strategy allowed for a comprehensive evaluation of the extract’s protective effects against PM_10_-induced oxidative stress and inflammation. Notably, the extract demonstrated concentration-dependent efficacy in reducing ROS levels, suppressing pro-inflammatory cytokines, and restoring genes associated with skin barrier function and extracellular matrix integrity.

Nevertheless, certain limitations must be acknowledged. The use of a single extract batch limited our ability to assess correlations between individual compound concentrations and specific bioactivities. Moreover, the network pharmacology analysis was constrained by the limited annotation of *Inula japonica* leaf-specific compounds in existing public databases, potentially affecting the completeness of the target-pathway associations.

Despite these constraints, the findings contribute novel insights into the pharmacological potential of *Inula japonica* leaf extract as a candidate for mitigating air pollution-induced skin damage. Further studies employing broader chemical libraries and targeted mechanistic assays are warranted to validate and expand upon these results.

## 5. Conclusions

In conclusion, Inula japonica leaf extract demonstrated protective effects against PM_10_-induced damage in human keratinocytes by reducing oxidative stress, suppressing inflammatory signaling pathways, and preserving epidermal barrier function. These effects were associated with decreased ROS production, inhibition of MAPK (ERK and p38) phosphorylation, and modulation of apoptosis- and inflammation-related proteins. Furthermore, the extract restored the expression of barrier-associated genes, including *loricrin*. UHPLC-MS analysis identified several compounds not previously reported in our earlier study, and network pharmacology analysis supported their potential involvement in the observed protective mechanisms. Collectively, these findings suggest that *Inula japonica* leaf extract holds therapeutic potential as a protective agent against air pollution-induced skin damage.

## Figures and Tables

**Table 1:** Primer sequences.

Genes	Sequence	
Human *cPLA2*	Forward Reverse	5′-GTGATGTGCCTGTGGTAG-3′ 5′-GGTGAGAATACAAGGTTGAC-3′
Human *KRT16*	Forward Reverse	5′-ATGCACAGTTCACTTTGCAGA-3′ 5′-CGCAAGAACAGCTCATTCTCG-3′
Human *Filaggrin*	Forward Reverse	5′-GCTGAAGGAACTTCTGGAAAGG-3′ 5′-GTTGTGGTCTATATCCAAGTGATC-3′
Human *Loricrin*	Forward Reverse	5′-GTGGGAGCGTCAAGTACTCC-3′ 5′-AGAGTAGCCGCAGACAGAGC-3′
Human *Involucrin*	Forward Reverse	5′-CAACTGGAGCTCCCAGAGCAGC-3′ 5′-AACACAGGCTGCTCCAGCTGC-3′

**Table 2:** Chemical profiling of the major flavonoids in INJA.

Peak	Identification	*t*_R_ (min)	Chemical Formula	Experimental *m*/*z*	MS/MS Fragment Ions (*m*/*z*)
**1**	Quercetin 3-*O*-glucuronide	11.68	C_21_H_18_O_13_	477.0660	301
**2**	Isoquercetin	11.88	C_21_H_20_O_12_	463.0870	301
**3**	Luteolin 3-*O*-glucuronide	12.06	C_21_H_18_O_12_	461.0714	85, 285
**4**	Isorhamnetin-3-*O*-glucoside	13.40	C_22_H_22_O_12_	477.1024	243, 314
**5**	Viscidulin III	15.56	C_17_H_14_O_8_	345.0605	164, 287, 315, 345
**6**	Luteolin	16.50	C_15_H_10_O_6_	285.0395	133, 151
**7**	Quercetin	16.57	C_15_H_9_O_7_	301.0344	107, 121, 139, 151, 178
**8**	4,5,7-Trihydroxy-3,6-dimethoxyflavone	17.88	C_17_H_14_O_7_	329.0656	164, 271, 299

**Table 3:** Constituents of *Inulae Flos* were selected from the Traditional Chinese Medicine Systems Pharmacology (TCMSP) database.

NO.	Name	OB (%)	DL
1	Chryseriol	35.85	0.27
2	Pratensein	39.06	0.28
3	Isorhamnetin	49.6	0.31
4	Beta-sitosterol	36.91	0.75
5	Amyrin Palmitate	32.68	0.3
6	Isoramanone	39.97	0.51
7	Tamarixetin	32.86	0.31
8	Inulicin	30.12	0.22
9	3-[(3aS,4R,5R,8aR)-4-hydroxy-5,7-dimethyl-3-methylene-2-oxo-4,5,8,8a-tetrahydro-3aH-cyclohepta[b]furan-6-yl]propyl acetate	73.35	0.22
10	[(3aR,4R,7aR)-5-[(1S)-4-acetoxy-1-methyl-butyl]-6-methyl-3-methylene-2-oxo-3a,4,7,7a-tetrahydrobenzofuran-4-yl] acetate	39.03	0.31
11	Azaleatin	54.28	0.3
12	Britanin	33.73	0.41
13	Epifriedelanol acetate	31.18	0.74
14	Melilotoside	36.85	0.28
15	Patuletin	53.11	0.34
16	Kaempferol	41.88	0.24
17	[(3S,4aR,6aR,6aR,6bR,8aR,12S,12aR,14aR,14bR)-4,4,6a,6b,8a,12,14b-heptamethyl-11-methylene-1,2,3,4a,5,6,6a,7,8,9,10,12,12a,13,14,14a-hexadecahydropicen-3-yl] Acetate	43.08	0.74
18	Luteolin	36.16	0.25
19	Quercetin	46.43	0.28

## Data Availability

All data generated or analyzed during this study are included in this published article.

## Abbreviations

The following abbreviations are used in this manuscript:

ATP5B	ATP synthase subunit beta
Bax	Bcl-2-associated X protein
ClueGO	Clustering with Gene Ontology
COX-2	Cyclooxygenase-2
cPLA2	Cytosolic Phospholipase A2
CYP2C8	Cytochrome P450 family 2 subfamily C member 8
CYP1A1	Cytochrome P450 family 1 subfamily A member 1
CYP1B1	Cytochrome P450 family 1 subfamily B member 1
DCFDA	2′,7′-Dichlorofluorescin Diacetate
DMEM	Dulbecco’s Modified Eagle Medium
DMSO	Dimethyl Sulfoxide
ECL	Enhanced Chemiluminescence
ERK	Extracellular signal-Regulated Kinase
FBS	Fetal Bovine Serum
GAPDH	Glyceraldehyde-3-Phosphate Dehydrogenase
HO-1	Heme Oxygenase-1
HCK	Hemopoietic cell kinase
HIBCH	3-hydroxyisobutyryl-CoA hydrolase
INJA	*Inula japonica* leaf extract
JNK	c-Jun N-terminal Kinase
KRT16	Keratin 16
KRT17	Keratin 17
KEGG	Kyoto Encyclopedia of Genes and Genomes
LC-MS	Liquid Chromatography–Mass Spectrometry
MAPKs	Mitogen-Activated Protein Kinases
MMP-9	Matrix Metalloproteinase-9
MMPs	Matrix Metalloproteinases
MCL1	Myeloid cell leukemia 1
NHKs	Normal Human Keratinocytes
PBS	Phosphate-Buffered Saline
PCR	Polymerase Chain Reaction
PIM1	Pim-1 proto-oncogene
PM_10_	Particulate Matter ≤10 μm in diameter
PPI	Protein–Protein Interaction
p53	Tumor protein p53
p-ERK	phosphorylated Extracellular Sig-nal-Regulated Kinase
p-JNK	phosphorylated c-Jun N-terminal kinase
p-p38	phosphorylated p38 mitogen-activated protein kinase
p-Akt	phosphorylated Protein Kinase B
qRT-PCR	Quantitative Real-Time PCR
ROS	Reactive Oxygen Species
SDS-PAGE	Sodium Dodecyl Sulfate Polyacrylamide Gel Electrophoresis
SLC2A2	Solute carrier family 2 member 2
STK17B	Serine/threonine kinase 17b
STRING	Search Tool for the Retrieval of Interacting Genes/Proteins
TCMSP	Traditional Chinese Medicine Systems Pharmacology
TBST	Tris-Buffered Saline with Tween 20
UHPLC	Ultra-High-Performance Liquid Chromatography
